# *Libidibia ferrea* Mature Seeds Promote Antinociceptive Effect by Peripheral and Central Pathway: Possible Involvement of Opioid and Cholinergic Receptors

**DOI:** 10.1155/2014/508725

**Published:** 2014-04-22

**Authors:** Luis Armando Sawada, Vanessa Sâmia da Conçeição Monteiro, Guilherme Rodrigues Rabelo, Germana Bueno Dias, Maura Da Cunha, José Luiz Martins do Nascimento, Gilmara de Nazareth Tavares Bastos

**Affiliations:** ^1^Laboratório de Neuroquímica Molecular e Celular, Instituto de Ciências Biológicas, Universidade Federal do Pará (UFPA), Rua Augusto Correa s/n, Guamá, 66075-900 Belém, PA, Brazil; ^2^Laboratório de Neuroinflamação, Instituto de Ciências Biológicas, Universidade Federal do Pará (UFPA), Rua Augusto Correa s/n, Guamá, 66075-900 Belém, PA, Brazil; ^3^Laboratório de Biologia Celular e Tecidual, Centro de Biociências e Biotecnologia, Universidade Estadual do Norte Fluminense Darcy Ribeiro (UENF), Avenida Alberto Lamego 2000, Parque Califórnia, 28013-602 Campos dos Goytacazes, RJ, Brazil

## Abstract

*Libidibia ferrea* (LF) is a medicinal plant that holds many pharmacological properties. We evaluated the antinociceptive effect in the LF aqueous seed extract and Lipidic Portion of *Libidibia ferrea* (LPLF), partially elucidating their mechanisms. Histochemical tests and Gas chromatography of the LPLF were performed to characterize its fatty acids. Acetic acid-induced abdominal constriction, formalin-induced pain, and hot-plate test in mice were employed in the study. In all experiments, aqueous extract or LPLF was administered systemically at the doses of 1, 5, and 10 mg/kg. LF aqueous seed extract and LPLF demonstrated a dose-dependent antinociceptive effect in all tests indicating both peripheral anti-inflammatory and central analgesia properties. Also, the use of atropine (5 mg/kg), naloxone (5 mg/kg) in the abdominal writhing test was able to reverse the antinociceptive effect of the LPLF, indicating that at least one of LF lipids components is responsible for the dose related antinociceptive action in chemical and thermal models of nociception in mice. Together, the present results suggested that *Libidibia ferrea* induced antinociceptive activity is possibly related to its ability to inhibit opioid, cholinergic receptors, and cyclooxygenase-2 pathway, since its main component, linoleic acid, has been demonstrated to produce such effect in previous studies.

## 1. Introduction

*Libidibia ferrea *(LF) is a plant used in folk medicine for therapeutical purposes. LF is a tree species from the Leguminosae family, one of the largest dicotyledon families with approximately 650 genus, that gathers more than 18,000 species. It is found along all tropical regions in Brazil but manly in the north and northeast areas [1].

Previous studies identified many pharmacological properties of LF, which explain its common use in folk medicine, for treatment of wounds, bruises, chronic cough, and asthma [2]. The fruits can be used to treat diabetes and to prevent cancer. Its extract proved in* in vivo *tests wound healing [3]. Roots are utilized as antipyretic and treatment of diarrhea. LF has also demonstrated anticancerogenic, antioxidant, wound repair, and DNA protection properties [4, 5]. Besides, the bark is used for enterocolitis, diabetes, and rheumatism treatment [6].

Thus, in the present study, we identified the main storage substances and their accumulation sites in* Libidibia ferrea* seeds, identified its main lipid components, and investigated the analgesic effect of LF on chemical and thermal models of nociception in mice and its possible mechanism of action.

## 2. Material and Methods

### 2.1. Plant Material

The plant was collected in Joanes, Salvaterra, Marajó Island, Pará, Brazil, during the year 2011 and classified by Dr. Silvane Tavares Rodrigues, Department of Botany, EMBRAPA. A voucher specimen (187419) was deposited in the IAN (which means Instituto Agronômico do Norte in portuguese) herbarium of the EMBRAPA (Belém, PA, Brazil). The specimen was identified as* Libidibia ferrea *or their synonym* Caesalpinia ferrea.* After collecting the material, seeds of* Libidibia ferrea *were separated for extraction.

### 2.2. Aqueous and Acetonic Extraction of* Libidibia ferrea*

To prepare the aqueous extract, fresh seeds weighing 50 g were cleaned in a water stream, extracted with 500 mL of Milli-Q water, and concentrated at a final volume of 14%.

For the preparation of the acetonic extract, seeds collected were dried up in natural temperature, powdered, and put into erlenmeyers. Then, acetone was added and the solution was incubated at one week. After that, the content of the erlenmeyer suffered a simple filtration using a filter paper; the liquid part separated was called the acetonic extract and it was stored at 4°C and after 5 days of storage, the acetonic extract was filtered again and the lipid portion of LF (LPLF) was used. The yield of lipidic portion was 20%.

### 2.3. Light Microscopy and Histochemical Tests

Histochemical tests were carried out using free-hand sections of newly collected material and included the Sudan IV test for lipids, Osmium tetroxide test for unsaturated lipids, and Nile blue sulphate test to neutral and acids lipids. Anatomical and histochemical descriptions were made with the aid of image analysis software (Axiovision) using an Axioplan Zeiss microscope.

### 2.4. Fatty Acid Analyses

Fatty acid analyses were made through Gas chromatography (GC), utilizing the official method AOCS Ce 1–62 [7]. The fatty acids methyl esters were prepared utilizing the AOCS Ce 1–62 method. The utilized equipment was the chromatograph VARIAN CP 3800 with flame ionization detector (FID). The compounds were separated using a CP WAX 52 CB column with 25 m length and 0.25 mm diameter; DF: 1, the operation conditions of the chromatographic analyses which were column programmed temperature: 80°C (1 min), injector temperature: 200°C, detector temperature: 250°C; carrier gas: helium, with a flow of 1.0 mL/min. Total routine time was 27 min. Fatty acid composition was calculated as a percentage of the total fatty acids present in the sample.

### 2.5. Animals

Swiss male mice (30–35 g) were obtained from the Evandro Chagas' Animal Resources Center, Belém, Pará, Brazil. They were randomly assigned to groups of 10 animals and maintained in plastic boxes, with food and water* ad libitum*, under a 12 h light/12 h dark cycle. The room temperature was maintained at 22 ± 1°C. The animals were acclimatized to the laboratory for at least 1 h before the experiments that were carried out between 8:00 and 13:00 h in order to avoid circadian influence. All experiments reported in this study were carried out in accordance with current guidelines for the care of laboratory animals and ethical guidelines for investigation of experimental pain in conscious animals. All efforts were made to minimize the number of animals used and their suffering.

### 2.6. Drugs, LPLF, and Aqueous Extract Administration

The present study utilized the following drugs: formalin, acetic acid, indomethacin, atropine, naloxone, celecoxib from Sigma Chemical Co., in St. Louis, MO, USA, and morphine hydrochloride from Cristalia-Brazil, São Paulo, Brazil. All drugs were dissolved in saline just before use, except celecoxib, which was dissolved in methylcellulose 0.5%. The extract was dissolved in saline and administered at doses of 1, 5, and 10 mg/kg, given intraperitoneally (i.p.). LPLF was given orally (p.o.). Morphine (10 mg/kg) and indomethacin (10 mg/kg) were used as positive control and were also administered by oral gavage. The negative control group was given saline, always in the same route of administration as the drug. The doses of all substances used in this work were selected based on previous results from our laboratory [8].

### 2.7. Antinociceptive Tests

#### 2.7.1. Abdominal Writhing by Intraperitoneal Injection of Acetic Acid

Abdominal contraction, induced by i.p. injection of acetic acid (1%), consisted of a contraction of the abdominal muscle together with a stretching of the hind limbs [9]. The animals were pretreated i.p. with indomethacin (10 mg/kg), used as positive control or with LF aqueous extract, i.p. (1, 5 or 10 mg/kg), 1 h before acetic acid administration. The control groups received the same volume, 0.9% of NaCl (10 mL/kg). After challenge, mice were placed in separate boxes and the number of abdominal constrictions was counted during the period of 30 min. Antinociceptive activity was expressed as the reduction in the number of abdominal constrictions.

#### 2.7.2. Formalin-Induced Licking

The procedure used was similar to that described previously [10]. Twenty microliters of 2.5% formalin solution (95% formaldehyde) were injected intraplantarly (i.pl.) under the ventral surface of the right hindpaw. Following intraplantar injection of formalin, each animal was immediately placed individually into a clear plexiglass cage (33 cm × 23 cm × 21.5 cm) and observed from 0 to 30 minutes. The amount of time spent licking the injected paw was timed with a chronometer and was considered as indicative of nociception. The initial nociceptive response normally peaked until 5 min after formalin injection (early phase) and 15–30 min after formalin injection (late phase), representing the tonic and inflammatory pain responses, respectively [11]. The animals were pretreated orally with the LPLF (1, 5, or 10 mg/kg) or indomethacin (10 mg/kg), which was used as positive controls, both 1 h beforehand. The control animals received the same volume of vehicle (saline, 10 mL/kg, i.p.) used to dilute the drug.

#### 2.7.3. Hot-Plate Test

The hot-plate test was used to measure the response latencies according to the method described previously [12]. One day before the test, mice were preselected on the hot plate at 55 ± 0.5°C. Animals showing a reaction time (latency for licking the hind feet or jumping) greater than 20 s were discarded. On the day of the experiment, animals were pretreated with saline (10 mL/kg, p.o.), morphine (10 mg/kg, p.o.), or LPLF (1, 5, or 10 mg/kg, p.o.) and 0, 0.25, 0.5, 1, 1.5, 2, and 2.5 h later, they were put on the heated surface of the plate at 55 ± 0.5°C. The time necessary for the initial response to the painful stimulus (taping of the paws, licking or jumping) was taken as defining nociceptive response. In order to minimize damage to the animals' paws, the cut-off time was 30 s.

### 2.8. Evaluation of the Mechanism of Action of the Seed LPLF

To assess the possible participation of the opioid, cholinergic, and inflammatory systems in the antinociceptive effect of the LF (10 mg/kg), mice were pretreated with naloxone (5 mg/kg, i.p.), atropine (5 mg/kg, i.p.), or celecoxib (5 mg/kg, i.p.), 30 min before the LPLF administration (10 mg/kg, p.o.). The nociceptive response was evaluated by the acetic acid-induced contortions and the evaluation of the mechanism of action was determined by the reversion of the LF seed LPLF antinociceptive effect.

### 2.9. Statistical Analyses

The results are presented as mean ± S.E.M., except the ID_50_ values (i.e., the doses of the aqueous extract or LPLF necessary to reduce response by 50% relative to control value) which are reported as geometric means accompanied by their respective 95% confidence limits. The ID_50_ values were calculated from at least three dosages of the extract and LPLF, determined by linear regression from individual experiments using appropriate software (SigmaPlot software, version 12.0). The statistical significance of differences between groups was made by one-way analysis of variance (ANOVA) followed by Tukey's multiple comparison test. *P* values less than 0.05 (*P* < 0.05) were considered to be significant.

## 3. Results

### 3.1. Analgesic Effect of* Libidibia ferrea* Aqueous Seed Extract on Chemical Model in Mice

**Figure 1:**
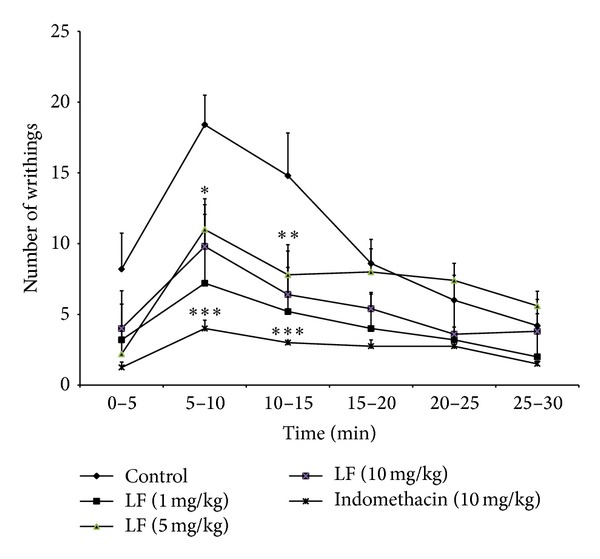
Effect of the aqueous extract of* Libidibia ferrea* (1, 5, and 10 mg/kg or indomethacin, 10 mg/kg), given i.p., on the writhing test in mice. The asterisks denote the significance levels in comparison with control groups, *P < 0.05, **P < 0.01, ***P < 0.001. In some cases, the error bars of the mean are hidden within the symbols.

*Libidibia ferrea* is a known popular used plant for treatment of different pain related injuries. In order to identify the possible analgesic effect of the LF aqueous seed extract, an abdominal writhing test was performed. Animalswere pretreated, 1 h beforehand, with the extract (given i.p., at the concentrations of 1, 5, and 10 mg/kg) and indomethacin (given p.o., 10 mg/kg). The aqueous extract caused a dose-related inhibition of 30 ± 12%, 47 ± 9%, and 73 ± 4%, respectively, and indomethacin demonstrated a 75 ± 6% inhibition; the calculated mean ID_50_ values for this effect was 5.99 mg/kg. Therefore, LF aqueous extract demonstrated an antinociceptive effect in chemical induced peripheral pain (Figure 1).

### 3.2. Histochemical Analyses of* Libidibia ferrea* Seeds

The plant seeds are one of the tree parts used for the analgesic treatment. So Histochemical tests of the seed were performed to identify its components.

**Figure 2:**
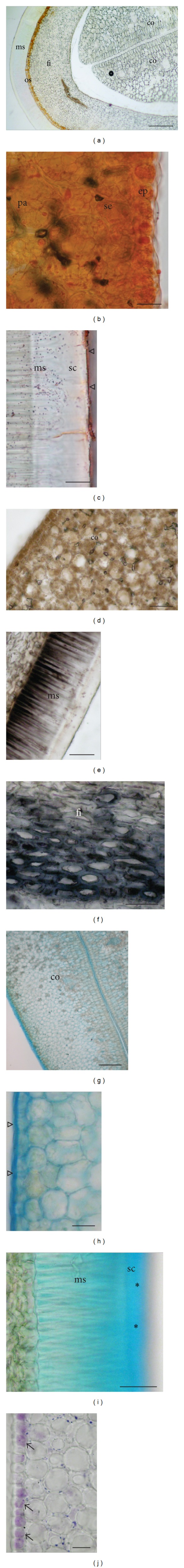
Histochemical tests in the longitudinal section of seeds of* Libidibia ferrea. *(a) Seed control showing the organization of main issues. (b) Sudan IV test that revealed the presence of lipids in the cotyledon and (c) in the seed coat cuticle. (d) Osmium tetroxide test showed that the lipids are mainly unsaturated and predominantly observed in the cotyledon, (e) in the basal portion of macrosclereids, and (f) in the fibers. (g-h) Nile blue sulphate test reveals the presence of neutral and acids lipids in the cuticle epidermis (open triangle) of cotyledons and (i) mainly in the subcuticular space (asterisks). (j) NADI reagent also revealed the presence of terpenoid only in the epidermal cells of cotyledons (arrows). ms: macrosclereid; os: osteosclereids; fi: fibers; pa: parenchyma; co: cotyledon; ep: epidermis of cotyledon; se: subepidermis of cotyledon; sc: subcuticular space. Scale bar: (a, g) = 200 *μ*m; (b-c, j) = 20 *μ*m; (d-e) = 100 *μ*m; (f, i) = 50 *μ*m; (h) = 25 *μ*m; (j) = 40 *μ*m.

*Libidibia ferrea* presented unitegumented seed composed of exotest epidermis with a thin cuticle layer; a subcuticular space and a palisade layer were formed by macrosclereids. A mesotest composed of osteosclereids and several layers of fibers, and an endotest composed of an inner surface; the space between the exotest and the cotyledon was occupied for endosperm (Figure 2(a)).

In the cotyledon, a great amount of the lipids were revealed with the Sudan IV test (Figure 2(b)) and a thin cuticle layer in the exotest epidermis (Figure 2(c)). Osmium tetroxide test revealed that most of the totality of lipids observed was mainly unsaturated and predominantly observed in the cotyledon (Figure 2(d)), in the macrosclereids, and in the thicker cell walls of the fibers (Figures 2(e)-2(f)).

Additionally, Nile blue sulphate test reveals the presence of the neutral and acid lipids in the cuticle and its cotyledonary adaxial epidermis and (Figure 2(g)) and mainly in the subcuticular space (Figures 2(h)-2(i)). Terpenoids were also observed only in the cotyledonary adaxial surface through the NADI reagent test (Figure 2(j)).

In summary,* Libidibia ferrea* has a large amount of lipids storage sites in different seed parts containing different lipids (unsaturated, neutral, and acid).

### 3.3. Gas Chromatography of the* Libidibia ferrea* Seed Lipid Portion

**Figure 3:**
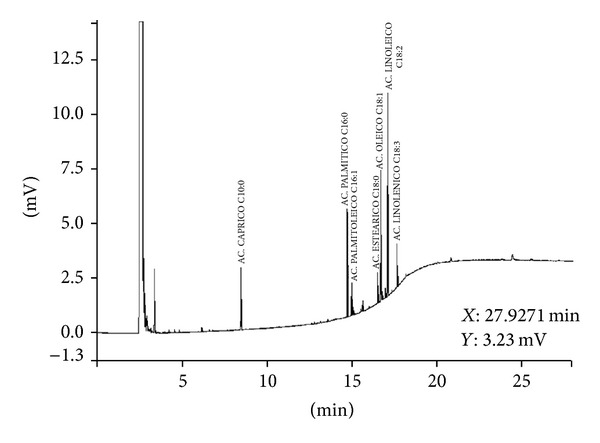
Methyl esters profile of the fatty acids in LPLF. GC with flame ion detection (FID). (1) Capric acid (C10:0); (2) palmitic acid (C16:0); (3) palmitoleic acid (C16:1); (4) stearic acid (C18:0); (5) oleic acid (C18:1); (6) linoleic acid (C18:2); (7) linolenic acid (C18:3).

GC identified the presence of different fatty acids in the LPLF (Figure 3). The percentages of the obtained values of GC for the chemical LPLF composition indicated a prevalence of unsaturated fatty acids as demonstrated in Table 1.

### 3.4. Mechanisms of Action of the Antinociceptive Effect of the LPLF

#### 3.4.1. Formalin Induced Licking

**Figure 4:**
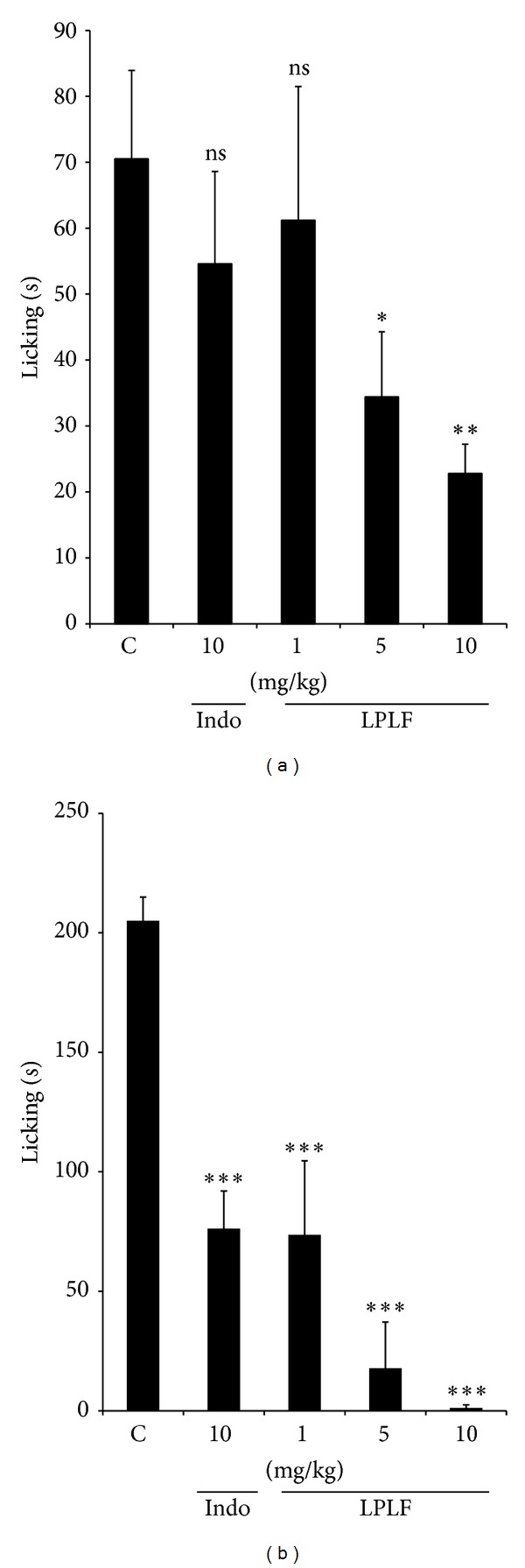
Effect of the LPLF (p.o.) and indomethacin (i.p.) given, against the early phase (0–5 min, panel (a)) or late phase (15–30 min, panel (b)) of formalin-induced nociception in mice. Each column represents the mean ± S.E.M. of 10 animals. Column C indicates the control values (animals injected with saline, 10 mL/kg) and the asterisks denote the significance levels in comparison with control groups, *P < 0.05, **P < 0.01, ***P < 0.001, compared to control group, Tukey's multiple comparison test.

LPLF could cause pain inhibition mainly by two neuronal pain mechanisms: central and peripheral. To investigate the analgesic effect in these different pain types, formalin was injected in the plantar left paw of pretreated mice and the time that they spent licking was counted. The maximal inhibition obtained in the early phase was 74 ± 2% and in the late phase 100%, while indomethacin caused significant inhibition (76 ± 7%) in the late phase (Figure 4). The calculated mean ID_50_ value for the early phase was 5.67 mg/kg and the late phase was 0.02 mg/kg; these data indicate that LPLF acts on both neurogenic and inflammatory pain.

#### 3.4.2. Hot Plate

**Figure 5:**
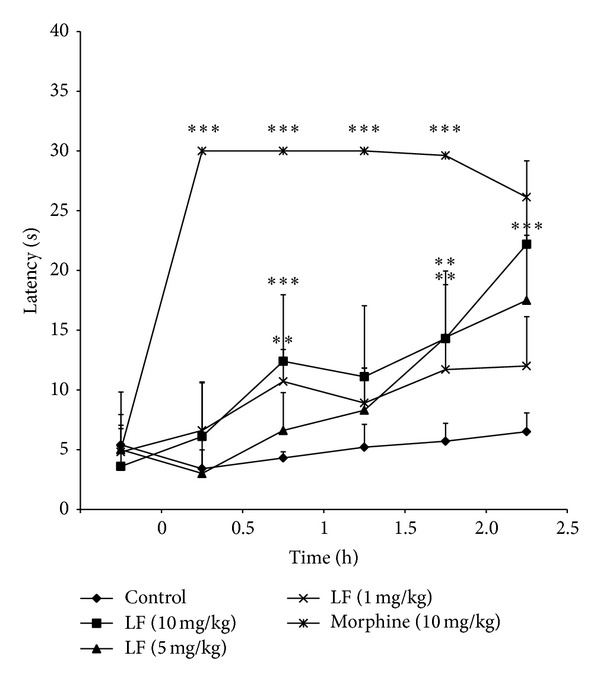
Effect of LPLF (1, 5, and 10 mg/kg) or morphine, (10 mg/kg), given orally, on the hot-plate test in mice. The asterisks denote the significance levels in comparison with control groups, *P < 0.05, **P < 0.01, ***P < 0.001. In some cases, the error bars of the mean are hidden within the symbols.

As previous data show* Libidibia ferrea *seed causes inhibition on neurogenic pain and to confirm this form of antinociception, a hot-plate test was performed. Pretreated mice were put on a heated plate and the time that took for them to jump, tap their foot, or lick their paw was counted. The LPLF caused an increase in the latency time of the animals in all analyzed periods at 2.5 h, indicating that LPLF could be promoting a central antinociceptive effect (Figure 5).

#### 3.4.3. Visceral Pain Induced by Acetic Acid and the Administration of Inhibitors

To evaluate by which central pain transmission pathway LPLF was acting on, mice were pretreated with naloxone and atropine (1.5 h prior the experiment) and then with LPLF (1 h prior the experiment). Naloxone and atropine were able to reverse LPLF antinociception. These data indicate that the LPLF might act on both opioid and cholinergic pathways.

**Figure 6:**
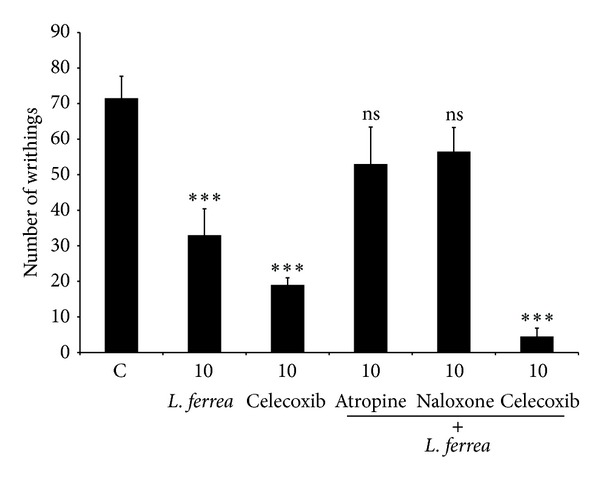
The effects of different antagonists on the antinociceptive activity of the LPLF in the acetic acid-induced contortions. Animals were pretreated with naloxone (5 mg/kg, i.p.), atropine (5 mg/kg, i.p.), or celecoxib (5 mg/kg, i.p.) 30 min prior to oral administration of the* Libidibia ferrea* seed oil (10 mg/kg, p.o.). The results are presented as the mean ± S.E.M (*n* = 6) of total writhings. ***P < 0.01 compared to control group, Tukey's multiple comparison test.

Also, the inhibition of peripheral inflammatory pain was further investigated with the administration of celecoxib (2 h prior the experiment) and then with LPLF (1 h prior the experiment), which increased the analgesic effect of the LPLF, demonstrating that LPLF probably causes an anti-inflammatory effect in a COX-2 select pathway (Figure 6).

## 4. Discussion

In the present study, we performed a phytochemical analysis (histology and GC of the LPLF), investigated LF analgesic properties, and tried to better elucidate LPLF mechanism of action. Results indicated that the seed's main lipids were Linoleic, Oleic, and Palmitic acid, stored in the cotyledon, macrosclereids, and thicker cell walls. Also, we discovered that aqueous extract and LPLF presented antinociceptive effects and that LPLF demonstrated peripheral and central forms of antinociception.

First, LF aqueous extract was tested in visceral nociception induced by acetic acid, which demonstrated a significant analgesic effect (Figure 1). This data corroborates with two other studies [13, 14]; however, both demonstrated lower analgesic effect. Carvalho et al. [14] used higher concentrations from 10 to 300 mg/kg of fruit crude aqueous extract and Freitas et al. [13] used 100 mg/kg of pods crude aqueous extract. In our study, lower concentrations (1–10 mg/kg) of seeds aqueous extract were used, which demonstrated a higher analgesic effect. The greater efficiency of the analgesic effect could be due to a greater presence of active compound(s) in the plant seeds.

Due to a significant layer of oil present in the seed extract used in the experiments, it was pondered if the analgesic agent(s) were present in this lipophilic portion. So the LPLF was isolated and GC evaluated its components, in order to identify analgesic related components, and histological assessments of the seeds were performed in order to indicate the storage site of such substances.

Histochemical seed analyses showed a mesotest composition and storage of endosperm between the exotest and cotyledon (Figures 2(b) and 2(c)), which is commonly the main seed storage tissues in the endospermic seeds, although LF presented a complete absorption of the endosperm during the embryogenesis [7, 15].

The lipids found in Sudan IV test (Figure 2(b)) occurred as oily bodies in different tissues of the seeds, such as cotyledon, endosperm, and embryo, since other tissues, beyond the cotyledon, can contribute to the storage of substances in the mature seed [15, 16].

Osmium tetroxide test revealed that most of the totality of lipids observed was mainly unsaturated and predominantly observed in the cotyledon (Figure 2(d)), in the macrosclereids, and in the thicker cell walls of the fibers (Figures 2(e)-2(f)).

Additionally, Nile blue sulphate test reveals the presence of the neutral and acid lipids in the cuticle and its cotyledonary adaxial epidermis and (Figure 2(g)) and mainly in the subcuticular space (Figures 2(h)-2(i)). Terpenoids were also observed only in the cotyledonary adaxial surface through the NADI reagent test (Figure 2(j)).

Additionally, findings in Nile blue sulphate test possibly confirm the presence of the triacylglycerols in the oily bodies (Figures 2(g) and 2(h)). The presence of terpenoids (Figure 2(j)), essential oil compounds found in seeds, flowers, leaves, roots, and wood of higher plants, could be related to previous detected LF antimicrobial activity, which has already been characterized as an LF property [17].

To identify the exact components present on the seed oily bodies and to further investigate the source of the antinociceptive effect, a Gas chromatography of the LPLF was performed (Figure 3). It identified the following components: capric, palmitic, palmitoleic, stearic, oleic, linoleic, and linolenic acid (Table 1).

In the analyses, linoleic acid (32.82%) was the most abundant fatty acid (Table 1). This seems to be common in this genus since* Caesalpinia sappan* [18] and* Caesalpinia echinata* [19] also have linoleic acid as their main fatty acid component with 31.6%, 31.3%, and 45.4%, respectively. But some other species such as* Caesalpinia bonducella* has hexadecadienoate (21.17%) as its prior fatty acid [20]. According to Mayworm and Salatino [21] most species from the Fabaceae family have predominance of the oleic acid and low quantities of fatty acids with more than 20 carbons, showing that LF differs from its family main lipid storage forms.

LF presented also a majority of unsaturated fatty acids which are represented as linoleic, oleic, linolenic, and palmitoleic acid, similar to* C. echinata* main components which are oleic, linoleic, and eicosanoic acid [19] and* C. bonducella* [20]. In the meantime,* C. sappan* had linoleic (31. 6%), oleic (27.3%), palmitoleic (18.76%), and linolenic acids (14.75%) [18].

The saturated fatty acids were found in a minor concentration in LF extract. The principal saturated fatty acid was the palmitic acid and in lower concentrations of capric and stearic acids. This data show similarity with* C. bonducella*, which had a 19.10% concentration of palmitic acid and other concentrations of saturated fatty acids. For* C. coriara* stearic acid was present in especially high amount (12.9%). Just like* C. echinata* that has steric acid (25.70%) as the main fatty acids and others like palmitic (13%), arachidic (4.10%), behenic (3.4%), and lignoceric (0.4%) acid in lower quantities [19, 22].

After these analyses, LPLF was used in subsequent antinociceptive tests (hot-plate and formalin-induced licking), which demonstrated a significant analgesic effect in both tests, confirming that the antinociceptive effect in the seeds extract could be attributed to at least one of LPLF components.

In formalin-induced licking test, LPLF demonstrated inhibition in both early and late phases. Data of the LPLF in the late phase indicate a strong inhibition in peripheral inflammatory induced pain (Figure 4(b)). According to the GC, the main components of the LPLF are linoleic and oleic acid. Omega-3 fatty acids, mainly linoleic acid, are used, in humans, as a supplementation for inflammatory joint pain [23, 24] and plants with high concentrations of linoleic acid usually have also analgesic effect [25].

When cells are exposed to certain stimuli such as inflammatory cytokines, lipopolysaccharide (LPS), and bacterial proteins, there is phosphorylation of I*κ*B (NF-*κ*B inhibitor) that leads to its degradation by proteasomes. This effect releases the NF-*κ*B from its inhibitory molecule and promotes translocation to the nucleus, which then binds to DNA, by the cis-acting *κ*B element, leading to transcription of several inflammatory genes such as COX-2 and iNOS [26].

External stimuli can also cause activation of certain Mitogen Activated Protein Kinases (MAPKs) such as MEKK1 (MAPKs/extracellular signal-regulated protein kinase (ERK) kinases (MEKs) Kinase1) that promotes the phosphorylation of I*κ*B*α* and IKK*β* complex, which are responsible for phosphorylating I*κ*B, therefore also promoting the release of NF-*κ*B and leading to inflammatory effects. On the other hand, the same stimuli could be activating MyD88, which activates the Akt pathway. Phosphorylation of Akt causes activation of NF-*κ*B, resulting in the increase of proinflammatory enzymes [27].

Our results corroborated with previous studies [28–30], which demonstrated that *α*-linoleic acid (5 and 10 mg/kg) showed a potent antinociceptive effect and concluded that *α*-linoleic acid downregulates inflammatory iNOS, COX-2, and TNF-*α* gene expressions through the blocking of NF-*κ*B and MAPKs activation in LPS-induced stimulated macrophages. Yasuda et al. [28] also demonstrated that 13-Hydroxy-10-oxo-trans-11-octadecenoic acid (13-HOA), one of the lipoxygenase metabolites of linoleic acid, suppresses the expression of LPS-induced proinflammatory genes in murine macrophages by disrupting MAPKs and Akt pathways.

Since anti-inflammatory drugs decrease the concentration of PGE_2_, which is responsible for activating and lowering the excitatory threshold of peripheral polimodal nociceptive neurons, anti-inflammatory drugs can cause a secondary analgesic effect. It is possible that the linoleic acid might be acting through this mechanism [31].

In addition, LPLF promoted an inhibition in the first phase of the formalin-induced licking test (10 mg/kg) and hot-plate test (10 mg/kg), indicating that LPLF also acts on a central nociception pathway (Figures 4(a) and 5). These results are more significant from those found by Carvalho et al. [14], which demonstrated a slight increase in the latency time (11.7 ± 0.8 s, using 100 mg/kg) of aqueous extract of fruit in hot-plate test. First phase of formalin tests predominantly evokes activity in A-delta fibers. Experimental results have indicated that substance P and bradykinin participate in this early phase [32]. It suggests that LF compounds could be promoting the antinociceptive effect in A-delta fibers.

To better characterize what peripheral and central pathways that LPLF was affecting, abdominal writhing test with the administration of different inhibitors was performed (Figure 6).

Reversal of the antinociceptive effect by opioid antagonist indicates a participation in central forms of antinociception. This effect corroborates with LPLF effect over K_ATP_ channels [33], since it is consistent with mechanism of action of opioids. Their receptors are bound to G-protein that regulates K^+^ channels and can also inhibit voltage gated Ca^2+^ channels in dorsal root ganglion neurons, which results in the inhibition of action potential propagation and neurotransmitter release, therefore preventing the stimuli from reaching the cortical nociceptive interpretation areas [34]; these forms of inhibition occur in various parts of the antinociception system, afferent neurons, spinal cord, midbrain, and thalamus [35]. Also, there is a regulation of central antinociception to peripheral nociception that LPLF might be activated, via PI3K*γ*/AKT/nNOS/NO signaling pathway [36]. Activation of *κ* opioid receptors promotes the activation of the PI3 K/Akt pathway that leads to activation of the nNOS and increasing the production of NO. This molecule then stimulates the cGMP/PKG that leads to the upregulation of K_ATP_ channels that increase the K influx leading the stabilization of the increased excitatory state present during the inflammation process [37]. However, further pharmacological studies are necessary to confirm this hypothesis.

Besides, our results show that atropine, given systemically, reversed the antinociceptive effect caused by LPLF, suggesting that antinociception detected for this compound depends, in part, on the cholinergic system. Several reports support a role for acetylcholine (ACh) in the inhibition and modulation of the transmission of nociceptive information [38, 39]. It has been demonstrated that the cholinergic system is involved in antinociceptive mechanisms, by (1) the activation of the GABAergic system [40]; (2) Muscarinic receptors (mAChR) mediation on spinal and (3) supraspinal sites [41, 42]; (4) inhibition enhancement of nicotinic receptors in the spinal dorsal horn [43]; (5) peripheral mAChR [44] or by interaction with the opioid system; however, this correlation is still not well elucidated.

To reduce the nociceptive information transition to the central nervous system (CNS), mAChRs when activated can potentiate the GABAergic tone through M(2), M(3), and M(4) subtypes [45, 46].

mAchRs are also present in the peripheral tissues and their activation suppresses pain impulses [47, 48].

In addition, antinociceptive agents, such as morphine, increase spinal release of ACh and endogenous ACh is an important mediator of the morphine analgesic effect.

In last, result demonstrating the effect of LPLF in abdominal writhing test with presence of celecoxib is due to a peripheral and central synergism, but it also might indicate a selective COX-2 anti-inflammatory action [49, 50].

Further pharmacological and chemical studies are necessary to confirm and to better characterize the exact mechanism(s) responsible for the antinociceptive action. Antinociceptive action demonstrated in the present study supports, at least partly, the ethnomedical uses of this plant and complements previous studies of this plant.

## 5. Conclusion

In summary,* Libidibia ferrea *seeds store linoleic, oleic, linolenic, palmitoleic, palmitic, stearic, and capric fatty acids, mainly in the cotyledon, macrosclereids, and thicker cell walls. The lipid portion of the* Libidibia ferrea* acetonic extract presented antinociceptive properties that are affected by inhibition of COX-2 and on cholinergic and opioid pathways.

## Figures and Tables

**Table 1:** Percentual composition of the fatty acids in *Libidibia ferrea* seed oil.

Fatty acids	Result (%)	Retention time (min)	Peak height (counts)
Insaturated			
Linoleic (C18:2)	32.82	8.43	9309
Oleic (C18:1)	21.79	14.67	6005
Linolenic (C18:3)	7.50	14.93	1992
Palmitolenic (C16:1)	5.48	16.45	1513
Saturated			
Palmitic (C16:0)	17.37	16.64	4960
Stearic (C18:0)	5.31	17.04	1414
Capric (C10:0)	9.73	17.59	2834

Total	100		28027
